# Supplementary material for: A Combination of an Angiotensin II Receptor and a Neprilysin Inhibitor Attenuates Liver Fibrosis by Preventing Hepatic Stellate Cell Activation
Source: Biomedicines. 2023 Apr 27;11(5):1295. doi: 10.3390/biomedicines11051295 (PMC10215948; doi:10.3390/biomedicines11051295)
Supplement: Supplementary file 1 [file biomedicines-11-01295-s001.zip › biomedicines-2312259-supplementary.pdf]

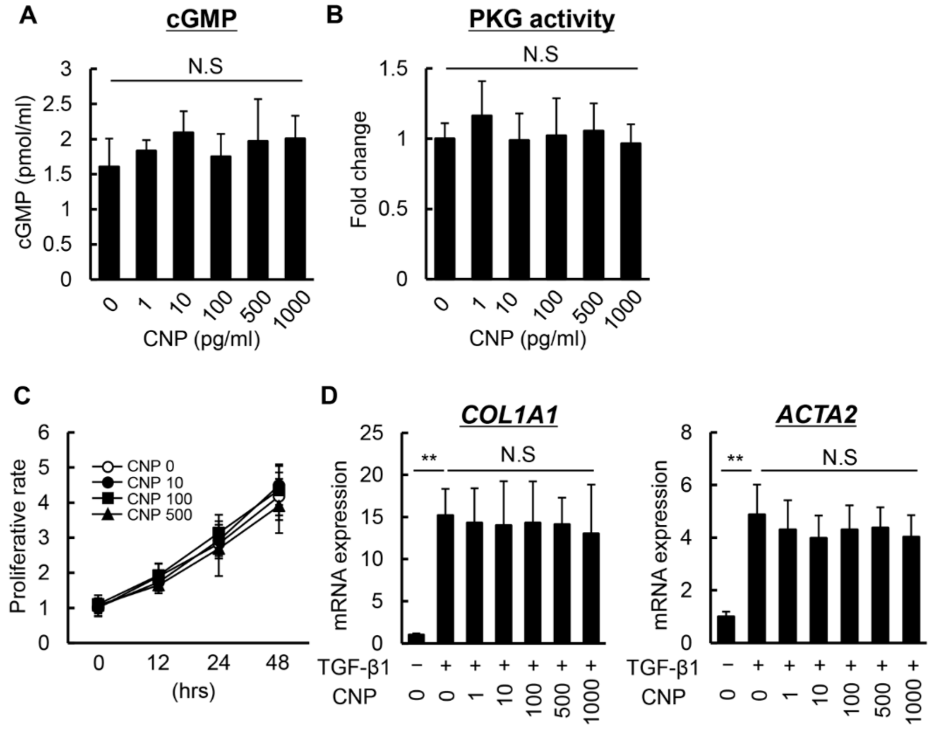

Supplementary Figure S1. (A) Intracellular cGMP levels in LX-2 cells incubated with CNP (0-1000 pg/ml). (B) Intracellular PKG activity in LX-2 cells incubated with CNP (0-1000 pg/ml). (C) Cell proliferation of LX-2 cells incubated with CNP (0-500 pg/ml). (D) Relative mRNA expression of *ACTA2* and *COL1A1* in LX-2 cells incubated with TGF-β1 (10 ng/ml), ANP (0, 100 and 500 pg/ml). *GAPDH* was used as internal control. Quantitative values are relatively indicated as fold changes to the values of non-treatment groups. Data are mean ± SD (n=8). ** p <0.01, indicating a significant difference between groups. N.S; not significant, CNP; C-type natriuretic peptide, PKG; protein kinase G.

Supplementary Table S1. List of primers used in q-PCR.

gene	Sense (5’-3’)	Antisense (5’-3’)
**Mouse**		
Acta2	CTGACAGAGGCACCACTGAA	CATCTCCAGAGTCCAGCACA
Col1a1	GAGCGGAGAGTACTGGATCG	GCTTCTTTTCCTTGGGGTTC
Tgfb1	TTGCTTCAGCTCCACAGAGA	TGGTTGTAGAGGGCAAGGAC
Agtr1	ATGGCTGGCATTTTGTCTGG	GTTGAGTTGGTCTCAGACAC
Gapdh	CTGCGACTTCAACAGCAACT	GAGTTGGGATAGGGCCTCTC
**Human**		
ACTA2	GAGACCCTGTTCCAGCCATC	TACATAGTGGTGCCCCCTGA
COL1A1	CCAAATCTGTCTCCCCAGAA	TCAAAAACGAAGGGGAGATG
MMP2	TGACGGTAAGGACGGACTC	ATACTTCACACGGACCACTTG
TIMP1	GCCCAGAGAGACACCAGAGAAC	CTATCAGCCACAGCAACAACAGG
TIMP2	GCAGGAGGAATCGGTGAGGTC	ACAGGCAAGAAGCAATGGCAAC
CTGF	ACCGACTGGAAGACACGTTTG	CCAGGTCAGCTTCGCAAGG
GAPDH	CCAAGGAGTAAGACCCCTGG	TGGTTGAGCACAGGGTACTT
